# Do positive childhood and adult experiences counter the effects of adverse childhood experiences on learned helplessness?

**DOI:** 10.3389/frcha.2023.1249529

**Published:** 2024-01-03

**Authors:** AliceAnn Crandall, Gladys Lopez Castaneda, Melissa J. Barlow, Brianna M. Magnusson

**Affiliations:** Department of Public Health, Brigham Young University, Provo, UT, United States

**Keywords:** learned helplessness, resilience, positive childhood experiences, positive adult experiences, adverse childhood experiences

## Abstract

**Introduction:**

Learned helplessness often arises when an individual feels that a challenging situation is inescapable. Childhood trauma can lead to feelings of learned helplessness in youth and adulthood. Resiliency theory suggests that positive experiences in childhood and adulthood may counteract traumatic experiences in childhood and reduce learned helplessness and promote learned optimism, the antithesis of learned helplessness. The purpose of this study was to examine the relationship of adverse childhood experiences (ACEs) with learned helplessness and optimism in adulthood and whether positive childhood and adult experiences (PCEs and PAEs) can lessen learned helplessness even in the presence of ACEs and promote greater learned optimism.

**Methods:**

The sample consisted of 435 adults who were recruited to participate in the study through Amazon Mechanical Turk (MTurk), a crowdsourcing recruitment service. Participants lived in the United States and were 18–56 years at the time of the study. Each participant completed a survey about their childhood and adulthood experiences and learned helplessness and optimism as an adult. Data were analyzed using structural equation modeling (SEM) in Mplus Version 7.

**Results:**

The learned helplessness scale had two factors which we termed learned helplessness and learned optimism. ACEs were associated with higher self-report of learned helplessness and lower learned optimism. When PCEs were added to the model, ACEs retained their relationship with learned helplessness but were no longer associated with learned optimism. PCEs were positively associated with learned optimism but were not associated with learned helplessness. PAEs were negatively correlated with learned helplessness and positively correlated with learned optimism. Learned optimism and learned helplessness in adults were inversely correlated.

**Discussion:**

Potentially traumatic experiences in childhood, as measured by ACEs, may lead to more learned helplessness throughout life. However, positive experiences in both childhood and adulthood can increase learned optimism, which was correlated with lower learned helplessness, even when childhood trauma was experienced.

## Introduction

Learned helplessness is the belief that one is unable to cope with adverse circumstances and the perception of uncontrollability over circumstances (1, 2). Individuals experiencing learned helplessness often feel that their behavior has no influence on the outcome (1). Learned helplessness is most commonly acquired through repeated experiences with adverse events that are perceived to be unavoidable or inescapable (2, 3). These experiences lead to a sense of powerlessness in confronting adversity and helplessness in changing circumstances, even when adopting new behaviors can produce positive outcomes (2).

Learned helplessness leads to deficits in three primary areas: motivation to change behavior, recognition of the association between behavior and outcome, and emotional well-being (2, 4). Several studies have concluded that there is a correlation between learned helplessness and mental illness, such as depression (2, 5, 6). One study explained that the Covid-19 pandemic could have contributed to feelings of learned helplessness and in turn depression among undergraduate students (6). Individuals experiencing learned helplessness may struggle to solve problems, feel powerless over their lives, and are more likely to experience increased stress, loss of self-confidence, and recover from mental illness at a slower rate (7).

The antithesis of learned helplessness is learned optimism. Learned optimism includes how one explains successes and justifies their past actions and prediction of future actions with a realistic but positive outlook and determined effort even in the face of challenge (8). Optimistic people regulate both their behavior and cognition, obtaining relevant information about rewards and risk to aid them in developing plans and making adjustments (9). Whereas learned helplessness contributes to worse health outcomes, learned optimism is associated with successful completion of treatment programs among children and adults (9) and lower mortality and engagement in more health promoting behaviors among older adults (10).

### Childhood trauma and learned helplessness

The term learned helplessness was coined by Martin Seligman. During an experiment conducted by Seligman (11), dogs were exposed to an inescapable electric shock. Later when the dogs were put in circumstances where the shock was escapable some dogs would attempt, initially, to avoid the shock but they would end up reverting back to dealing with the electric shock rather than trying to escape it. Roughly two-thirds of the dogs who received the inescapable electric shock did not learn to escape it in situations where it was possible to get away from it; the other third did learn to get away from it and avoid it (11).

Like the dogs in Seligman's experience, people can also develop learned helplessness from being exposed to events that may appear, at least initially, inescapable. However, unlike dogs, learned helplessness in people can be more complex (12). For example, learned helplessness may not be generalizable to all situations that an individual is in. Furthermore, humans may see a circumstance as changeable but not changeable by themselves (12). Childhood trauma is an example of an event with repeated exposure that can lead to learned helplessness. Adverse childhood experiences (ACEs) are potentially traumatic events composed of early childhood maltreatment, family dysfunction, and other stressors that are associated with an increased development of negative health behaviors in adulthood, such as higher levels of impulsivity, hostility, and suicidal ideation (13). There is usually no way for a child who is experiencing ACEs to escape or control the situation they are in. A variety of studies show that learned helplessness and ACEs are correlated. A study conducted by Gomez et al. (14) showed that emerging adults who were coming out of the foster care system commonly reported higher learned helplessness when compared to other emerging adults. Another study found a correlation between violence between siblings (e.g., sexual contact, manipulation, threats, coercion, repeated acts of aggression) and learned helplessness (15). While another study demonstrated that children who live in poverty for a prolonged amount of time have higher levels of learned helplessness and more mental illness (16). Other studies suggest that stress from unemployment can lead to learned helplessness, whereas some studies show that employment can lead to lower levels of learned helplessness (17, 18). This demonstrates that childhood trauma and ACEs can impact learned helplessness in both childhood and adulthood.

### Positive experiences and learned helplessness and optimism

Recent research indicates that positive childhood experiences (PCEs) and positive adult experiences (PAEs) may exert independent and opposite influence on adult wellbeing compared to ACEs (19–21). PCEs and PAEs, like ACEs, are cumulative measures of advantageous experiences in childhood (before age 18 years) and adulthood, respectively (20, 22). These experiences include having a variety of healthy relationships (e.g., relationships with family members, peers/friends, neighbors, teachers, the community, and so forth) and having structure, stability, and positive meaning in life (20, 22).

Children who feel as though they have some control over their future, are more likely to feel empowered, make positive decisions, and overcome setbacks (23). These are children with learned optimism. However, children who believe they have no power are more likely to succumb to helplessness, increased stress, and develop the inability to cope when new setbacks arise in their lives (23). Research on learned optimism is much more limited than studies on learned helplessness, and most research has focused on optimism itself rather than optimism that is learned. One study of a clinical sample examined both learned helplessness and learned optimism. Using a sample of 25 hospitalized children with a variety of diagnoses, each child was to attempt to solve a puzzle within nine minutes. The children were separated into two groups: positive reinforcement and negative. The children in the positive group would be rewarded with a soda at the end of each session and the children in the negative group would receive a punishment. These problem-solving sessions would end once the child no longer wanted to attempt solving the puzzle. It was found that the children in the positive reinforcement group had more perseverance, higher motivation, and superior problem-solving skills than those in the non-positive reinforcement group (24). Although this study has not been replicated in a non-clinical sample, it exemplifies the theory that the more children persevere in a challenging task in the face of positive reinforcement, the more they realize they are capable of handling new challenges and feel empowered to overcome them, moving from an internal dialogue of “Nothing I do matters” to “I can make a difference (23).

To our knowledge, there are no studies that have examined the effects of PCEs on reducing learned helplessness in adulthood, with or without trauma. Furthermore, in the event that ACEs were high and PCEs were low, little is known about whether PAEs can serve as turning points for reducing learned helplessness in adults who experienced trauma. Studies on childhood factors associated with better learned optimism in adulthood are limited. However, the compensatory model of resiliency theory (25) may serve as a theoretical framework for understanding these relationships. The compensatory model of resiliency theory suggests that positive and adverse experiences will exert independent but opposing influence on an outcome, and in some cases the inclusion of the positive experience may neutralize some of the negative effects of adversity on an outcome (25). The compensatory model of resiliency theory differs from the protective factors model in that it does not assess whether the positive factor may moderate the relationship between adversity and an outcome. Prior research indicates that PCEs are likely to be compensatory factors but have not been shown to consistently work as a moderator or protective factor (26). A compensatory factor is any type of positive behavior (emotional support, healthy eating, exercise, etc.) that produces healthy outcomes in the face of trauma, allowing for individuals to prevent the development of negative risks and overcome barriers in development. For example, in a study conducted by Zimmerman, Steinman & Rowe (1998), it was found that children with higher levels of parental support had less violent behavior than children without parental support, demonstrating the compensatory effect of parental support in counteracting the development of negative behaviors of violence (27).

### Aims and hypotheses

Drawing on the compensatory model of resiliency theory (25), the primary aims for the paper included: (1) Are ACEs associated with higher learned helplessness and lower learned optimism? (2) Are PCEs associated with lower learned helplessness and higher learned optimism even in the presence of ACEs? and (3) Are PAEs correlated with lower self-reported learned helplessness and higher learned optimism? We hypothesized that ACEs would be associated with higher rates of learned helplessness, lower learned optimism, and fewer self-reported PAEs. However, consistent with the compensatory model of resiliency theory, we hypothesized that the relationship between ACEs and learned helplessness would decrease when PCEs were added to the model and that PCEs would be associated with more learned optimism. We also hypothesized that PAEs would be correlated with lower learned helplessness.

## Materials and methods

Participants for this study were recruited via Amazon Mechanical Turk (MTurk). Mturk is a crowdsourcing web recruitment service. Researchers (called “requesters” on MTurk) are able to recruit participants (called “workers on MTurk) who meet the requester's defined characteristics based on their MTurk profile information. Previous research has found that MTurk samples have good generalizability to national samples (28, 29).

In the current study, the final sample included 435 adults, ages 18–56 years, who lived in the United States. Workers who were registered on MTurk and who were eligible for the study (adults ages 18 years and older living in the United States) were able to see a description of the study. Those interested completed informed consent and a 15-min survey administered on the Qualtrics platform. After completing the survey, a quality check was conducted to ensure that participants who completed the survey were not bots, that they met age and location criteria, and that they had passed questions meant to check for attention to survey items (e.g., “for this item select strongly agree.”). Approximately 10% of those who took the survey were rejected during the quality check. Participants were given a $2.00 credit in their MTurk account.

### Measures

#### Learned helplessness and learned optimism

Learned helplessness and learned optimism were measured using the 20-item Learned Helplessness Scale (30). Response options were on a 4-point Likert scale ranging from *Strongly Disagree* to *Strongly Agree*. Sample items included “My behavior seems to influence the success of a workgroup” and “No matter how hard I try, things never seem to work out the way I want them to.” The authors of the scale identified five factors in the scale (30). However, two of the factors only had two items, which would have led to an under-identified model. As such, we conducted an exploratory factor analysis (EFA) to determine the most appropriate factor structure for our sample. Prior to conducting the EFA, we determined factor loading cutoffs (items < .40 would be dropped) and model fit cutoffs (RMSEA < .08 and CFI > .90 indicated acceptable fit) (31, 32). We also considered whether the items retained and factor structure fit with theory (31, 33) on learned helplessness. According to Quinless and colleagues (30), the developers of the learned helplessness measure, items should measure three dimensions: (1) Examination of whether learned helplessness occurs on a continuum of internality vs. externality attributional style; (2) The ability to distinguish learned helplessness across a variety of situations vs. helplessness in specific situations only; and (3) Determination of whether learned helplessness deficits consistently occur over time. The first three factors of the Quinless five-factor structure of the learned helplessness scale measured the three dimensions of learned helplessness listed above; factors four and five measured items theoretically related to but tangential to learned helplessness theory (30). Other researchers have noted that the 20-items fit a unidimensional (one-factor) factor structure of the learned helplessness scale (34). In the current sample, Cronbach's alpha for a unidimensional scale was .86. However, the one-factor model had poor fit in exploratory factor analysis (EFA), and eigen values and the EFA results suggested a two-factor model, with items that were negatively worded loading on one factor (9 items; factor named learned helplessness) and items that were positively worded loading on a second factor (10 items; factor named learned optimism). Item four was dropped due to a low factor loading (<.40). The item that was dropped came from factor five of the Quinless factor structure, one of the factors tangential to the learned helplessness theory (30). As such, we determined that dropping this item did not affect the theoretical integrity of the measure.

#### Positive adult experiences

A 15-item positive adult experiences scale was developed and validated as a part of the larger study (22). Response options were 1 = yes, and 0 = no. Sample items included “In the past two weeks, I have talked to somebody outside of my family about my feelings” and “I feel like I belong in my community.” Cronbach's alpha for the current sample was .77. Items were summed for a cumulative PAEs score ranging from 0 to 15.

#### Adverse childhood experiences

The 11-item ACEs module from the Centers for Disease Control and Prevention's Behavioral Risk Factor Surveillance System Survey (35) was used to measure adverse childhood experiences. Response options were dichotomous (1 = yes; 0 = no). In accordance with guidelines from the CDC on the coding of ACEs (36), substance abuse and sexual abuse items were combined so that there were a total of eight possible ACEs. Responses were summed for a total ACEs score ranging from 0 to 8. Sample items included “When you were growing up, during your first 18 years of life…Did you live with anyone who was depressed, mentally ill, or suicidal?” and “When you were growing up, during your first 18 years of life… Did you live with anyone who served time or was sentenced to serve time in a prison, jail, or other correctional facility?” In the current sample, the ACEs measure had good internal reliability (*α* = .80).

#### Positive childhood experiences

To measure PCEs, we used the 10-item Benevolent Childhood Experiences scale (20). We also included the first three items from the Positive Childhood Experiences scale (19). This resulted in a 13 item PCE measure, which has indicated good reliability in prior studies (37). Response options were dichotomous (1 = yes; 0 = no). A cumulative score of PCEs was created by summing the 13 items, with possible scores ranging from 0 to 13 PCEs. Sample items included “When you were growing up, during your first 18 years of life…Did you feel that your family stood by you during difficult times?” and “When you were growing up, during your first 18 years of life…Did you have at least one caregiver with whom you felt safe?” Internal reliability for the sample was good (*α* = .84).

#### Controls

To account for potential differences in reporting by gender, age, and socioeconomic status, we included the following demographics as controls in the model: gender (1 = female; 0 = male), age (in years), education (1 = Bachelor's degree or higher; 0 = less than a Bachelor's degree), race (1 = White; 0 = non-White), and marital status (1 = married; 0 = not married).

#### Data analysis

Data were cleaned and item means and distributions were examined in Stata 17. Structural equation modeling (SEM) was conducted in Mplus version 7 to examine the study aims. The measurement model was established using confirmatory factor analysis (CFA). The two learned helplessness subscales (learned optimism and learned helplessness) were included in the model as latent variables. Next, two structural models were run. The first structural equation model regressed learned optimism, learned helplessness, and positive adult experiences on adverse childhood experiences. Next, positive childhood experiences were added to the model with adverse childhood experiences. ACEs and PCEs were allowed to be correlated, as were positive adult experiences, learned optimism, and learned helplessness. The models controlled for gender, age, education, race, and marital status by regressing all covariates of interest (learned helplessness, learned optimism, PAEs, ACEs, and PCEs—in the second model) on these controls. Model fit for the CFA and structural models was examined using the comparative fit index (CFI), scores greater than .95 were considered excellent fit and above .90 adequate fit, and the root mean square error of approximation (RMSEA), with scores less than .08 considered adequate fit and less than .05 considered good fit. Model fit cutoffs are important in SEM as they demonstrate the model's fit to the data and are sensitive to the sample size (38). Therefore, adequate fit indicates that the sample was sufficient and that the model and data were a good fit. All models were estimated using a robust weighted least squares approximation, which is appropriate for categorical data. Missing data were minimal (<1% across all variables with no missing data on learned helplessness/optimism items) and accounted for using full information maximum likelihood (FIML).

## Results

The average participant age was 37 years old. Slightly less than half (48%) of the sample reported their sex as female. The majority (74%) of the sample reported their race as White, and 69% had a Bachelor's degree. Nearly one-third (30%) of the sample had a household income less than $40,000/year. Table 1 includes the full descriptive statistics for the sample. Over one-third of the sample (35%) reported four or more ACEs. On average, participants had 2.5 ACEs, 10.7 PCEs, and 12.5 PAEs. Supplementary Table S1 includes bivariate correlations between all study covariates and controls.

**Table 1:** Descriptive statistics, *N *= 435.

Sample characteristic	*M* (*SD*)
Age	36.59 (9.40)
Female (%)	47.59
Married (%)	56.32
White (%)	73.79
Household income < $40,000/Year (%)	29.89
Bachelor's degree or higher (%)	68.51
Currently employed (%)	88.74
ACEs score (R: 0–8)	2.53 (2.29)
≥ 4 ACES (%)	34.94
PCEs score (R: 0–13)	10.66 (2.86)
PAEs score (R: 0–15)	12.51 (2.67)

### Measurement model

Model fit for the CFA of the measurement model (learned helplessness and learned optimism) was adequate (CFI: .975; RMSEA: .063). Standardized factor loadings ranged from .71 to .87 for learned helplessness and from .51 to .81 for learned optimism.

### The effects of childhood experiences on learned helplessness and optimism

**Figure 1:**
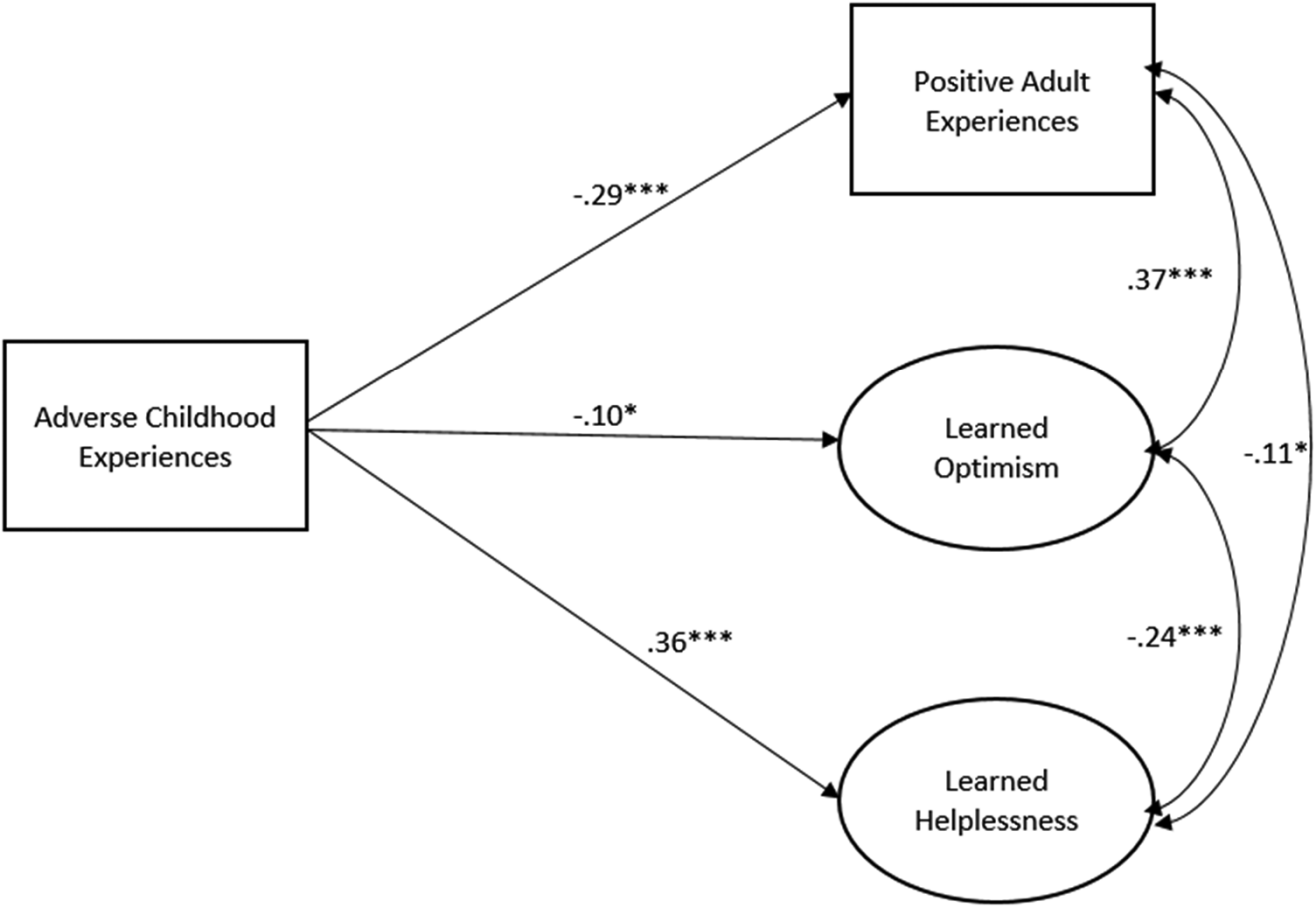
Structural equation model of ACEs and adult learned helplessness/optimism and PAEs, *N* = 435. Model Fit: RMSEA: 0.054; CFI: 0.0966. Model controls for gender, age, education, race, and marital status. Only significant paths shown.

**Figure 2:**
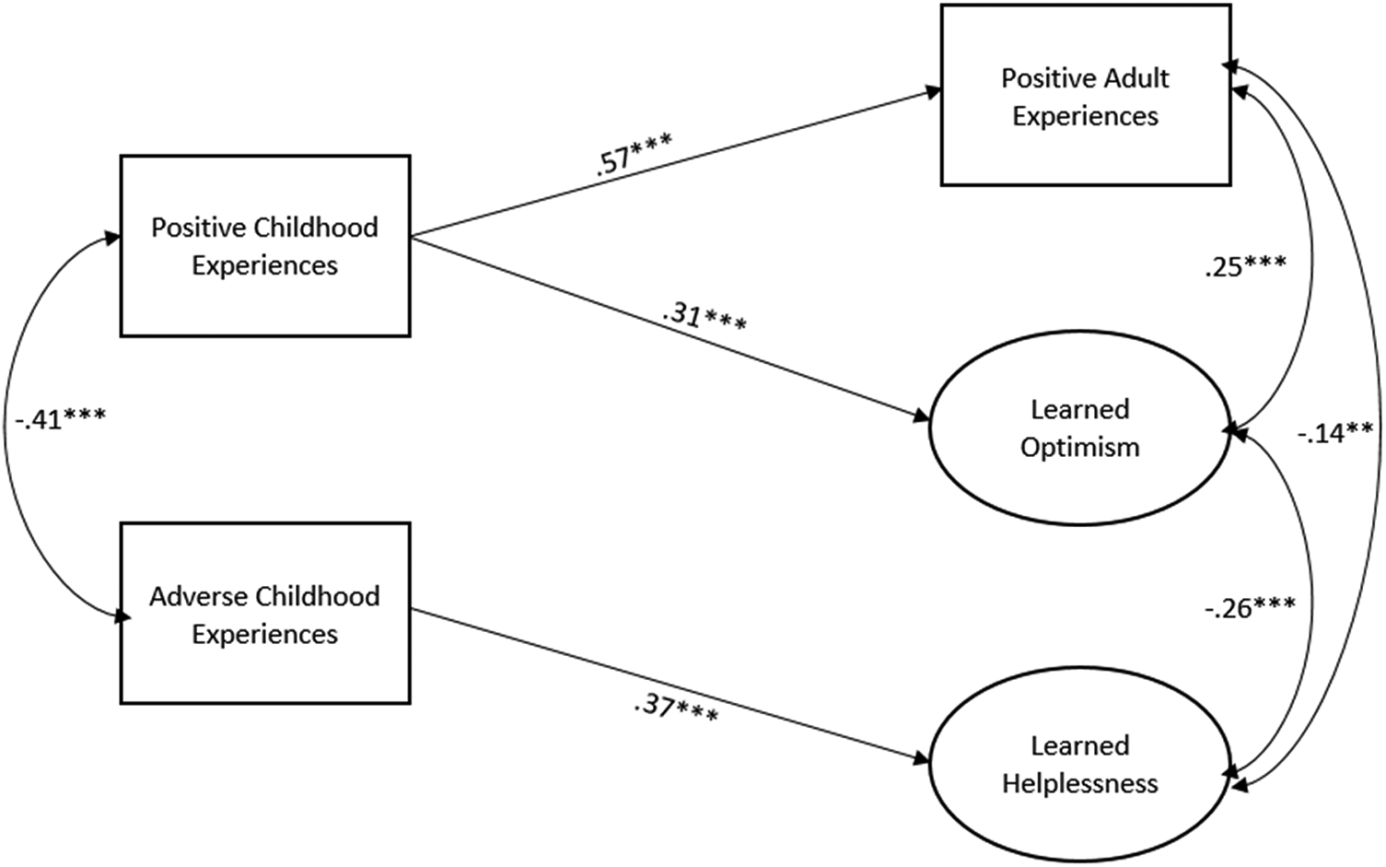
Structural equation model of association between childhood experiences and adult learned helplessness/optimism and PAEs, *N* = 435. Model Fit: RMSEA: 0.052; CFI: 0.0959. Model controls for gender, age, education, race, and marital status. Only significant paths shown.

In the model without positive childhood experiences (Figure 1; all betas standardized), ACEs were associated with fewer PAEs, increased learned helplessness, and lower learned optimism. When PCEs were added to the model (Figure 2; all betas standardized), ACEs were only associated with higher learned helplessness. Positive childhood experiences were associated with higher PAEs and higher learned optimism. Learned helplessness was negatively correlated with learned optimism whereas PAEs were negatively correlated with learned helplessness and positively correlated with learned optimism. The Supplemental File includes full results including the relationship between the controls and covariates of interests for Figures 1, 2 (see Supplementary Tables S2, S3 respectively).

## Discussion

The purpose of this study was to assess whether ACEs and PCEs were associated with learned helplessness and learned optimism and whether PAEs, controlling for ACEs and PCEs, were correlated with lower self-reported learned helplessness and higher learned optimism. The results largely substantiated the hypotheses. Specifically, ACEs were associated with higher self-reported learned helplessness and lower learned optimism (hypothesis 1). When PCEs were added to the model, ACEs were no longer associated with learned optimism (hypothesis 2), but ACEs were still associated with higher learned helplessness (the standardized betas were similar with and without PCEs in the model) which was contrary to hypothesis 2. PCEs were associated with more PAEs and higher learned optimism. PAEs were correlated with lower reported learned helplessness and higher learned optimism (hypothesis 3).

### Adversity and learned helplessness

Given the breadth of research on trauma and learned helplessness (e.g. (11, 13–15), it is not surprising that we found a relationship between ACEs and learned helplessness in adulthood though it ran contrary to our hypothesis that with PCEs and PAEs in the model ACEs would no longer be associated with increased learned helplessness. However, in this study we did not examine the timing and duration of the ACEs and PCEs, which all may affect the extent to which ACEs lead to learned helplessness (39–41). The timing and duration of ACEs are crucial, in that early and prolonged negative experiences in childhood can have detrimental effects on an individual's perception of control and agency (39). As such, even if positive experiences occur later in childhood or in adulthood, they may not be enough to undo the psychological damage caused by ACEs that were prolonged and/or that occurred early in childhood. Individual differences in psychological resilience may also play a significant role, as some individuals may be better able to overcome the effects of ACEs while others may be more vulnerable to developing learned helplessness, regardless of the presence of positive experiences in their adult life (40). Another factor that may influence the association of ACEs and learned helplessness is the development of coping mechanisms. If coping mechanisms developed in childhood are passive or avoidant, the effects of ACEs are more likely to contribute to mental health problems such as helplessness, anxiety, and depression (41).

### Positive experiences and learned optimism

It is promising and consistent with resiliency theory (25) that positive experiences, both in childhood and adulthood, were associated with reduced learned helplessness and increased learned optimism irrespective of ACEs. This suggests that it is never too late for individuals to learn optimism through surrounding themselves with a variety of positive relationships and meaningful routines. PCEs and PAEs are both cumulative measures of a variety of positive experiences, particularly positive relationships with peers, a variety of adults, neighbors, and the community. A few positive experiences may not produce the same results. Rather, based on our findings it appears that it is an accumulation of a variety of positive experiences, particularly healthy relationships, that makes the difference. In prior research, high means for PCEs (19–21) and PAEs (22) have been found, similar to the means in the current study. In research examining PCEs and ACEs with mental health, those with below the mean PCEs had higher odds for depression and other health problems than those with moderate or low levels of PCEs (37).

Positive experiences are particularly important for helping people to increase their feelings of and belief in their efficacy or learned optimism and may have less of an impact on reducing learned helplessness. The relationship between PAEs and PCEs with learned optimism held true while holding ACEs constant, suggesting that with or without trauma positive experiences increase learned optimism. Optimistic people believe they have control over their actions and, to some extent, the outcomes. For adults who have experienced high ACEs or even trauma in adulthood, research indicates that optimism may lead to posttraumatic growth (42). Posttraumatic growth increases appreciation of life, awareness of personal strengths, awareness of new possibilities and choices, and religious faith and spiritual understanding; it also aids in creating closer and more meaningful relationships with others (43, 44). In the current study, we did not examine posttraumatic growth, and an important future next step for research is to examine the degree to which PCEs and PAEs may lead to posttraumatic growth.

### Learned helplessness factor structure

It is worth noting that other studies that included the learned helplessness scale used different factor structures. The original authors used a five-factor model (30), and others have suggested using the scale as a single factor of learned helplessness (34). Our results suggest that a five-factor model may not be identifiable in data analysis, and a two-factor model may allow for the examination of both learned helplessness and its antithesis of learned optimism. The differences in factor structure may be due to the method used (e.g., in this paper we conducted a CFA and examined both factor loadings and model fit, whereas other papers have focused more on exploratory factor analysis) or could be due to differences in sample characteristics across time [the original study was also conducted among adults but was published in 1988 (30)].

### Application to practice

As much as preventing and reducing trauma will always be the goal, population efforts to increase PCEs and PAEs may be more realistic and effective than complete eradication of ACEs. Some existing programming may serve as blueprints for increasing positive experiences for children and adults. For example, the HOPE (health outcomes from positive experiences) framework is designed to support positive relationships for children and their parents and may help to reduce ACEs (45). The Family Checkup (https://fcu.uoregon.edu/), Triple P (Positive Parenting Program; https://www.triplep.net/glo-en/home/), Communities That Care (http://communitiesthatcare.net), and the Strengthening Families Program (https://www.extension.iastate.edu/sfp10-14/) are examples of family-level programming that may reinforce PCEs and PAEs and have been certified as model plus, model, or promising practice programs by Blueprints for Healthy Youth Development (46). Further research on these programs is needed to examine to what degree they help to increase PCEs and PAEs, especially in the presence of high ACEs.

### Limitations and strengths

Limitations of the study include that the data were cross-sectional, as an online, convenience sample the results may not be generalizable to some populations, and all measures were based on self-report. Longitudinal data are needed to better assess the role of PCEs and PAEs over time in sustaining reductions in learned helplessness even among those with high childhood trauma and examining learned optimism over time. Additionally, it will be important to replicate the study in more nationally representative samples and diverse samples. Additionally, it will be important to understand if results are similar for in-person samples vs. online samples. For example, the MTurk sample in the current study might introduce inequities (e.g., due to computer literacy) that may not be accounted for by ensuring sociodemographic representation alone. It is worth noting that more than one-third of the current sample experienced high ACEs, whereas only 12.5% of the CDC-Kaiser ACEs Study reported four or more ACEs (47). Finally, although PCEs, ACEs, PAEs, and learned helplessness and optimism are typically measured by self-report, there are likely some ways of measuring learned helplessness/optimism through proxy measures that are more objective, such as dropout or completion rates at challenging tasks.

Despite these limitations, the findings help to fill a gap in the literature of looking at positive experiences in both childhood and adulthood and their association with learned helplessness and optimism even in the face of trauma. Future studies can build on the research in this study to examine changes in learned helplessness and optimism over time, in more diverse samples, and with both objective and self-reported measures of learned helplessness and optimism. The results provide an impetus for more programming at the individual, family, and community level to increase positive experiences throughout the life course leading to more learned optimism and the potential for posttraumatic growth in the face of high ACEs and other trauma.

## Data Availability

The datasets presented in this article are not readily available because we do not have IRB approval to share it outside of approved study personnel. Requests to access the datasets should be directed to AC at ali_crandall@byu.edu.
