# Supplementary material for: Do positive childhood and adult experiences counter the effects of adverse childhood experiences on learned helplessness?
Source: Front Child Adolesc Psychiatry. 2024 Jan 3;2:1249529. doi: 10.3389/frcha.2023.1249529 (PMC11732079; doi:10.3389/frcha.2023.1249529)
Supplement: Supplementary file 1 [file Table1.docx]

**Supplemental File**

| Supplemental Table 1. Correlation Matrix of All Study Covariates and Controls | | | | | | | | | | |
| --- | --- | --- | --- | --- | --- | --- | --- | --- | --- | --- |
|  | Female | Age | BS Degree | White | Married | ACEs | PCEs | PAEs | Learned Helplessness | Learned Optimism |
| Female | 1.00 |  |  |  |  |  |  |  |  |  |
| Age | .06 | 1.00 |  |  |  |  |  |  |  |  |
| BS Degree | .00 | -.13* | 1.00 |  |  |  |  |  |  |  |
| White | .24** | .13* | -.04 | 1.00 |  |  |  |  |  |  |
| Married | .09 | .12* | .43*** | .25** | 1.00 |  |  |  |  |  |
| ACEs | .07 | -.06 | .01 | .18** | .22*** | 1.00 |  |  |  |  |
| PCEs | -.04 | .02 | .22*** | -.02 | .14* | -.39*** | 1.00 |  |  |  |
| PAEs | .12 | .04 | .21*** | .10 | .30*** | -.24*** | .61*** | 1.00 |  |  |
| Learned Helplessness | .02 | -.18*** | .30*** | .11 | .31*** | .40*** | -.08 | -.11* | 1.00 |  |
| Learned Optimism | .03 | -.03 | .34*** | .08 | .45*** | -.03 | .35*** | .44*** | -.10* | 1.00 |

**p* < .05. ***p* < .01. ****p* < .001.

| Supplemental Table 2. Adjusted model without PCEs (associated with Figure 1). | | | | |
| --- | --- | --- | --- | --- |
|  | ACEs | PAEs | Learned Helplessness | Learned Optimism |
| ACEs | -- | -.29*** | .36*** | -.10* |
| Female | .04 | .08 | -.01 | .01 |
| Age | -.09 | -.00 | -.16** | -.06 |
| BS Degree | -.05 | .10* | .17** | .17** |
| White | .10* | .06 | .03 | .03 |
| Married | .18** | .25*** | .14** | .32*** |

**p* < .05. ***p* < .01. ****p* < .001.

| Supplemental Table 3. Adjusted model with PCEs (associated with Figure 2). | | | | | |
| --- | --- | --- | --- | --- | --- |
|  | ACEs | PCEs | PAEs | Learned Helplessness | Learned Optimism |
| ACEs | -- | -- | -.06 | .37*** | .03 |
| PCEs | -- | -- | .57*** | .02 | -.31*** |
| Female | .04 | -.04 | .10* | -.01 | .02 |
| Age | -.09 | .04 | -.00 | -.16** | -.06 |
| BS Degree | -.05 | .17*** | .02 | .17** | .12* |
| White | .10* | -.02 | .05 | .03 | .02 |
| Married | .18** | .07 | .17*** | .14** | .28*** |

**p* < .05. ***p* < .01. ****p* < .001.
